# Gallbladder agenesis diagnosed intra-operatively: a case report

**DOI:** 10.1186/1752-1947-4-285

**Published:** 2010-08-23

**Authors:** Sachin Malde

**Affiliations:** 1 Department of Surgery, Fairfield General Hospital, Rochdale Old Road, Bury, BL9 7TD, UK

## Abstract

**Introduction:**

Agenesis of the gallbladder is a rare congenital anomaly occurring in 13 to 65 people of a population of 100,000. The rarity of the condition, combined with clinical and radiologic features that are indistinguishable from those of more common biliary conditions, means that it is rarely diagnosed preoperatively, and patients undergo unnecessary operative intervention.

**Case presentation:**

This case report describes the case of a 79-year-old symptomatic Caucasian man who underwent laparoscopic cholecystectomy for suspected choledocholithiasis despite imaging studies raising suspicion of gallbladder agenesis. Intra-operatively, the diagnosis of gallbladder agenesis and associated common bile duct stones was made.

**Conclusion:**

The preoperative diagnosis of this rare condition is difficult to make. However, with advances in biliary tract imaging and with heightened awareness of this anomaly, fewer patients will need to undergo unnecessary operative intervention. The authors review the different imaging modalities available to help diagnose this condition and highlight the importance of being aware of this rare anomaly to avoid an operation that carries a high risk of iatrogenic injury.

## Introduction

Isolated agenesis of the gallbladder is a rare congenital anomaly that results from failure of the cystic bud to develop *in utero*. Since its first description by Lemery in 1701, a number of cases have been published, with a reported incidence of 0.01% to 0.06% [1]. Patients are usually asymptomatic, and the diagnosis is commonly made as an incidental finding during abdominal surgery or at autopsy [2]. It is estimated that 25% to 50% of patients will develop common duct stones at some point, and 23% will eventually become symptomatic, usually in the fourth or fifth decade [3,4]. Symptoms mimic those of common biliary conditions such as cholecystitis, and routine investigations fail to distinguish between gallbladder agenesis and other conditions such as cholecystitis with cystic duct obstruction or an atrophic gallbladder. Combined with the rarity of the condition, the diagnosis is infrequently made preoperatively, and so the patient undergoes unnecessary operative intervention. Intraoperatively, the risk of iatrogenic injury is higher, and so the associated morbidity of the procedure is greater [5].

Despite recent advances in biliary tract imaging, the pre-operative diagnosis of gallbladder agenesis remains elusive. Here this case report describes a case of symptomatic gallbladder agenesis with common duct stones diagnosed at laparotomy and discuss the utility of the various imaging modalities that are currently available to attempt to diagnose this condition.

## Case presentation

A 79-year-old Caucasian man presented to the clinic with reduced appetite, unintentional weight loss of approximately 6 kg, and a history of fatty food intolerance. He denied any abdominal pain or febrile episodes, and physical examination was unremarkable. Biochemical investigations, however, revealed deranged liver-function tests with total bilirubin, 66 μmol/L; ALT, 122IU/L; ALP, 274IU/L; and gamma GT, 864IU/L.

An abdominal ultrasound showed a dilated common bile duct (CBD) with stones inside it. The gallbladder was not visualized, but strong echoes with acoustic shadowing were seen, suggesting a contracted gallbladder. A computed tomography (CT) scan revealed stenosis of the proximal CBD and dilated intra- and extra-hepatic bile ducts. Furthermore, it showed a small pseudocystic structure that was assumed to be a shrunken gallbladder.

**Figure 1:**
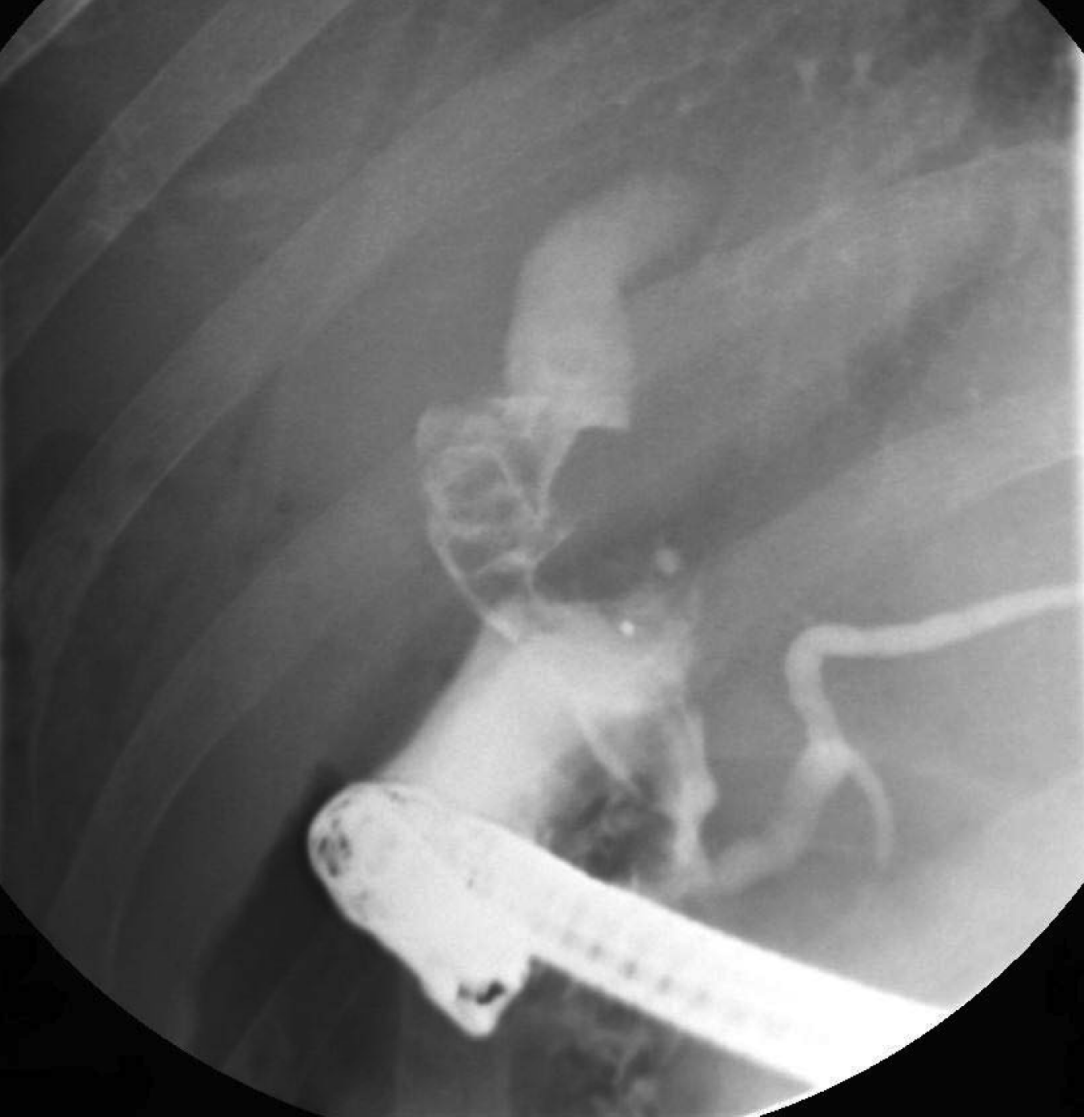
Pre-operative endoscopic retrograde cholangiopancreatography (ERCP) showing a dilated common bile duct (CBD) with stones and absence of the gallbladder.

The patient proceeded to endoscopic retrograde cholangiopancreatography (ERCP), which showed multiple stones (the largest measuring 1.5 cm) in the CBD, which could not be removed, and so a stent was inserted (see Figure 1). The gallbladder was not visualized, but this was thought to be the result of insufficient contrast. A repeated ERCP a few months later was reported as having cleared the CBD of all stones; the stent was removed, but the gallbladder had still not been visualized. As he had developed some intermittent right upper quadrant pain over this time, he was listed for a laparoscopic cholecystectomy for presumed choledocholithiasis.

**Figure 2:**
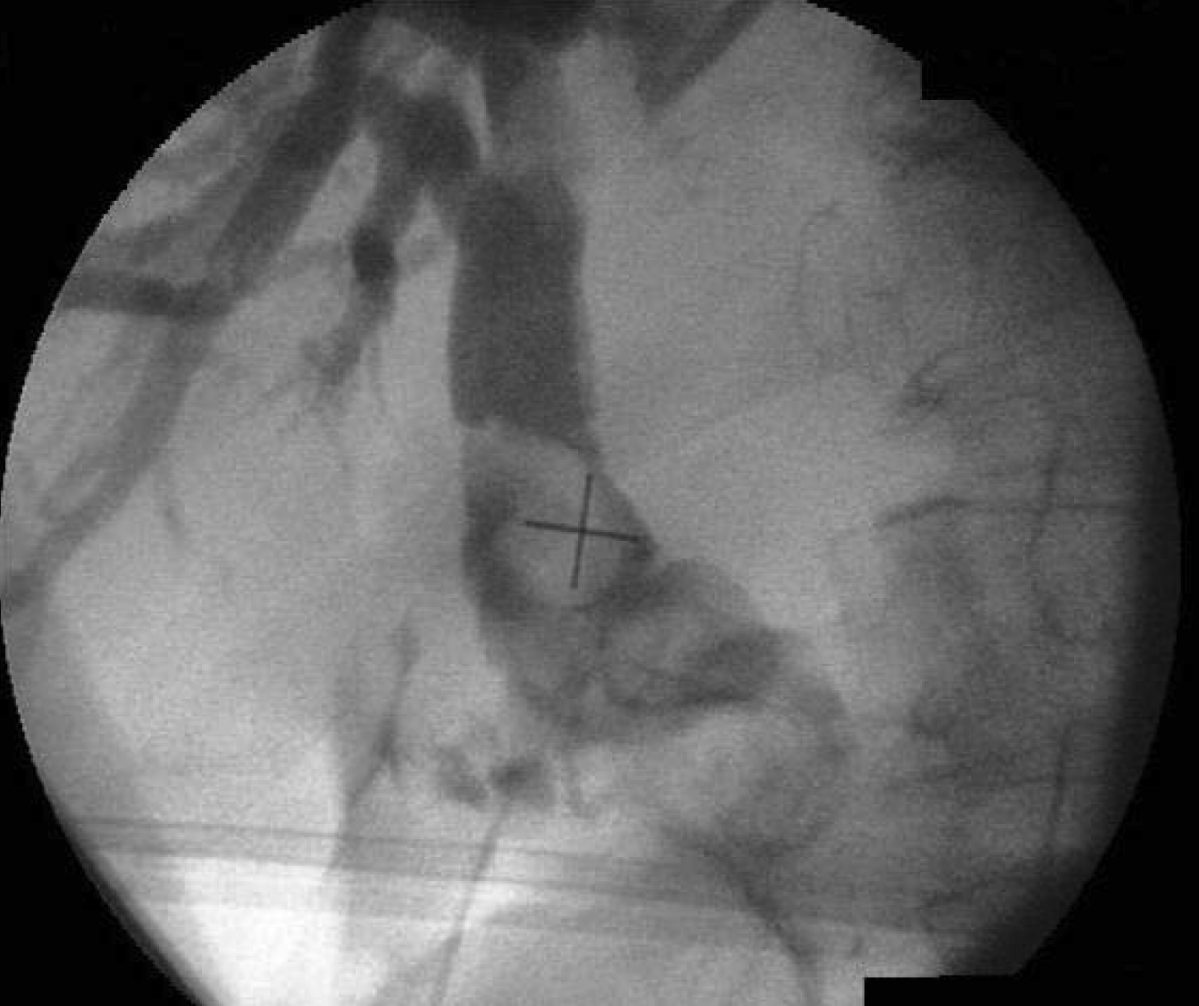
Intra-operative cholangiogram confirming common bile duct (CBD) stones and agenesis of the gallbladder.

At laparoscopy, a small fibrous remnant was seen in the gallbladder fossa, but the gallbladder could not be found despite an extensive search of all possible ectopic sites. Conversion to an open procedure and on-table cholangiogram revealed a dilated CBD and confirmed gallbladder agenesis (see Figure 2). The CBD was explored, numerous stones removed, and a T-tube was inserted.

Post-operatively, he made an uneventful recovery, and remains symptom free.

## Discussion

The liver, gallbladder, and biliary system begin to develop early in the fourth week of intrauterine life as a ventral outgrowth from the caudal part of the foregut. This hepatic diverticulum divides into two parts as it grows, one representing the primordium of the liver, and the other, the primordium of the gallbladder and cystic duct. By the seventh week, vacuolation occurs, and the gallbladder and cystic duct develop a lumen. Failure of this developmental process at any stage results in agenesis of the gallbladder [6], whereas inappropriate migration of the gallbladder primordium will result in an ectopic gallbladder. Potential sites of ectopic gallbladder are intra-hepatic, left-sided, beneath the posterior inferior surface of liver, between the leaves of the lesser omentum, within the falciform ligament, retroperitoneal, retrohepatic, or in the retropancreatic and retroduodenal areas [7].

Clinically, three groups of presentation of gallbladder agenesis have been described [1]: (1) asymptomatic (an incidental finding at laparotomy for another reason) (35%), (2) symptomatic (50%), (3) in children with multiple fetal anomalies (such as tetralogy of Fallot and agenesis of the lungs) who die in the perinatal period (15% to 16%).

Symptomatic patients commonly present with right upper quadrant pain, dyspepsia, jaundice, fatty food intolerance, or nausea, but these symptoms are indistinguishable from those of other common biliary tract conditions, making diagnosis difficult. It has previously been suggested that the pathophysiology of symptoms in gallbladder agenesis is similar to that of the post-cholecystectomy syndrome, and it is thought that the causes of pain include biliary dyskinesia and choledocholithiasis [8].

Management options for this symptomatic group include smooth muscle relaxants, and if this fails, sphincterotomy [9]. Importantly, laparotomy is not indicated if this benign condition is diagnosed pre-operatively. Therefore, if it is diagnosed pre-operatively, the patient is spared operative intervention. However, failure of the currently available imaging modalities to differentiate accurately between agenesis of the gallbladder and other biliary diseases, combined with the lack of awareness of this condition, has meant that the majority of patients undergo laparotomy, with its associated morbidity.

In the 1960s, Frey [10] suggested that the diagnosis of agenesis of the gallbladder could be made only at laparotomy after having searched for, and excluded, an ectopic gallbladder in the sites mentioned earlier, after which an intra-operative cholangiogram should be undertaken to confirm the diagnosis. However, the development of different imaging techniques over the years has led people to question the necessity of operative intervention for the diagnosis of this rare condition [11].

The usual initial investigation for patients presenting with right upper quadrant pain is an abdominal ultrasound. It has been suggested that the absence of the ultrasonographic features of the WES triad (visualization of the gallbladder wall, the echo of the stone, and the acoustic shadow) and the double-arc shadow should raise suspicion of gallbladder agenesis as the diagnosis [2]. However, the limitations of this investigation are well known. It has a reported sensitivity of 95% in diagnosing gallstones but is dependent on many factors, including the operator's experience and the examination conditions. Furthermore, shadowy opacities thought to represent gallstones could actually be due to intestinal gas artefact, periportal tissue, or subhepatic peritoneal folds, leading to false-positive findings [12]. Gallbladder agenesis cannot be reliably differentiated from the shrunken, contracted gallbladder of chronic cholecystitis, and this is the most frequent radiologic report seen in patients later found to have agenesis of the gallbladder. In these cases, it has been suggested that further imaging should be obtained before operative intervention to increase the accuracy of the diagnosis [1,11].

Hepatobiliary scintigraphy scans (such as ^99m^Tc-HIDA) are promising in the diagnosis of various gallbladder anomalies, including agenesis. However, nonvisualization of the gallbladder also typifies cystic duct obstruction secondary to acute cholecystitis, and so symptoms are more often attributed to this condition [13].

Computed tomography (CT) scanning and ERCP are further techniques that can be used to diagnose agenesis of the gallbladder. In combination with ultrasound, ERCP increases the likelihood of successful diagnosis. However, non-visualization of the gallbladder is commonly attributed to an obstructed cystic duct, anatomic variations, or technical errors (as in our case), and agenesis of the gallbladder is considered the least likely explanation. Recent literature suggests that CT and ERCP are useful postoperative modalities, if gallbladder agenesis is suspected at laparoscopy [11]. In this instance, laparotomy and extensive dissection to look for the missing gallbladder are discouraged, and instead, postoperative imaging is advised [14].

If gallbladder agenesis is suspected pre-operatively, endoscopic or laparoscopic ultrasound has been shown to be effective in confirming the suspicion [15]. However, these investigations are not currently widely available, thereby limiting their utility. Magnetic resonance cholangiopancreatography (MRCP) is being increasingly used in cases of diagnostic uncertainty to confirm the diagnosis. This test is noninvasive and is not affected by biliary stasis.

**Figure 3:**
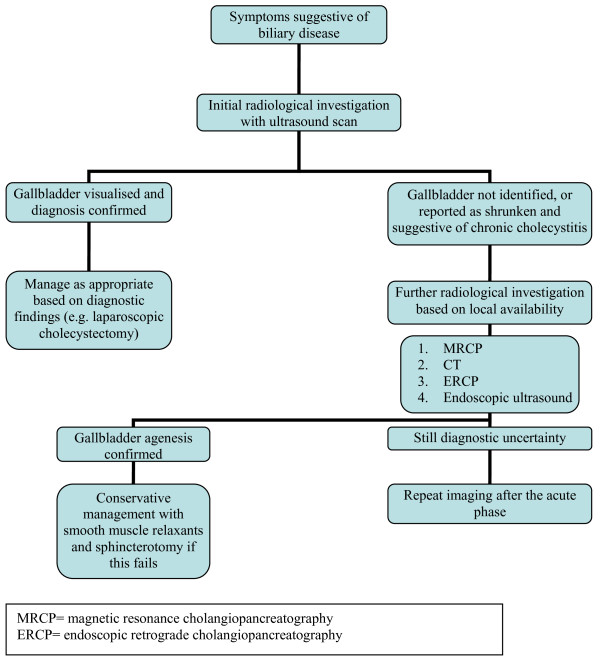
Suggested decisional tree for the investigation of suspected gallbladder agenesis.

A lack of awareness of this condition among surgical, gastroenterologic, and radiologic staff was the main reason for operative intervention in this case, and the subsequent conversion to an open procedure. Despite a suggestive ultrasound, CT, and ERCP, the diagnosis was still not made, and the patient underwent cholecystectomy for presumed gallstones. This highlights the need for greater appreciation of agenesis of the gallbladder as a cause of biliary symptoms, especially when initial radiologic tests suggest an absent gallbladder. A suggested decisional tree for the investigation of suspected gallbladder agenesis has been devised (see Figure 3) in an attempt to identify this rare condition pre-operatively, thereby preventing the unnecessary operative intervention seen in this case.

## Conclusion

In conclusion, agenesis of the gallbladder is a rare but well-recognized congenital anomaly, the management of which is conservative. However, clinical and radiologic features mimic those of more common biliary conditions, and so patients frequently undergo unnecessary operative procedures. With the newer minimally invasive radiologic techniques, this situation can largely be avoided if awareness of this condition is improved. Pre-operative MRCP should be considered in cases in which ultrasound suggests nonvisualization of the gallbladder, and surgeons should maintain a low threshold for further investigation before any decision to operate. A conservative approach with follow-up imaging is advocated in cases of doubt to avoid unnecessary operations. In cases that are diagnosed at laparoscopy, the author agrees with the other authors that further procedures should be abandoned, and the patient should undergo post-operative investigation with the radiologic modalities already described, to prevent the morbidity of conversion to an open procedure.

## Consent

Written informed consent was obtained from the patient for publication of this case report and accompanying images. A copy of the written consent is available for review from the journal's Editor-in-Chief.

## Competing interests

The author declares that they have no competing interests.

## References

[B1] Bennion RS Thompson JE Tompkin RK Agenesis of the gallbladder without extrahepatic biliary atresia Arch Surg 1988 123 1257 1260 3052366 10.1001/archsurg.1988.01400340083014

[B2] Kabiri H Domingo OH Tzarnas CD Agenesis of the gallbladder Curr Surg 2006 63 104 106 10.1016/j.cursur.2005.04.018 16520109

[B3] Peloponissios N Gillet M Cavin R Halkic N Agenesis of the gallbladder: a dangerously misdiagnosed malformation World J Gastroenterol 2005 11 6228 6231 16273658 10.3748/wjg.v11.i39.6228 PMC4436648

[B4] Wilson JE Deitrick JE Agenesis of the gallbladder: case report and familial investigation Surgery 1986 99 106 109 3510477

[B5] Cabajo CM Martin del Olmo JC Blanco AJ Atienza SR Gallbladder and cystic duct absence: an infrequent malformation in laparoscopic surgery Surg Endosc 1997 11 483 484 10.1007/s004649900397 9153182

[B6] Turkle SB Swanson V Chandrasoma P Malformations associated with congenital absence of the gallbladder J Med Genet 1983 20 445 449 10.1136/jmg.20.6.445 6655671 PMC1049178

[B7] Sherson ND The absent adult gallbladder Aust N Z J Surg 1970 39 225 261 10.1111/j.1445-2197.1970.tb05601.x 5268977

[B8] Toouli J Geenen JE Hogan WJ Dodds WJ Arndorfer RC Sphincter of Oddi motor activity: a comparison between patients with common bile duct stones and controls Gastroenterology 1982 82 111 117 7053322

[B9] Chopra PJ Hussein SS Isolated agenesis of the gallbladder Saudi Med J 2003 24 409 410 12754546

[B10] Frey C Bizer L Ernst C Agenesis of the gall bladder Am J Surg 1967 114 917 926 10.1016/0002-9610(67)90418-7 4862849

[B11] Grandhi TM El-Rabaa SM Agenesis of the gall bladder and cystic duct: laparoscopic diagnosis Int J Gastroenterol 2005 4 1

[B12] Serour F Klin B Strauss S Vinograd L Agenesis of gallbladder revisited laparoscopically Surg Laparosc Endosc 1993 2 144 146 8269237

[B13] Gad MA Krishnamurthy GT Glowniak JV Identification and differentiation of congenital gallbladder abnormality by quantitative technetium-99m IDA cholescintigraphy J NuclMed 1992 33 431 434 1740714

[B14] Balakrishnan S Singhal T Grandy-Smith S El-Hasani S Agenesis of the gallbladder: lessons to learn JSLS 2006 10 517 519 17575771 PMC3015765

[B15] Chan FL Chan JK Leong LL Modern imaging in the evaluation of hepatolithiasis Hepatogastroenterology 1997 44 358 369 9164502

